# Outcomes 'out of africa': the selection and implementation of outcome measures for palliative care in Africa

**DOI:** 10.1186/1472-684X-11-1

**Published:** 2012-01-06

**Authors:** Julia Downing, Steffen T Simon, Faith N Mwangi-Powell, Hamid Benalia, Barbara A Daveson, Irene J Higginson, Richard Harding, Claudia Bausewein

**Affiliations:** 1 Honorary Professor Palliative Care, Makerere University, Kampala, c/o PO Box 72518, Kampala. Formerly Deputy Executive Director, African Palliative Care Association, Kampala, Uganda; 2 Department of Palliative Medicine and Clinical Trials Unit (BMBF 01KN1106), University Hospital Cologne, Kerpener Strasse 62, 50924 Köln, Germany; 3 African Palliative Care Association, PO Box 72518, Kampala, Uganda; 4 King's College London, Department of Palliative Care, Policy and Rehabilitation, Cicely Saunders Institute, Denmark Hill, London, SE5 9PJ, UK

**Keywords:** Palliative care, Online survey, Outcomes, Outcome measurement, Palliative care Outcome Scale (POS), APCA African POS, Research, Africa

## Abstract

**Background:**

End-of-life care research across Africa is under-resourced and under-developed. A central issue in research in end-of-life care is the measurement of effects and outcomes of care on patients and families. Little is known about the experiences of health professionals' selection and implementation of outcome measures (OM) in clinical care, research, audit, or teaching in Africa.

**Methods:**

An online survey was undertaken of those using outcome measures across the region, as part of the PRISMA project. A questionnaire addressing the use of OMs was developed for a similar survey in Europe and adapted for Africa. Participants were sampled through the contacts database of APCA. Invitation emails were sent out in January 2010 and reminders in February 2010.

**Results:**

168/301 invited contacts (56%) from 24 countries responded, with 78 respondents having previously used OM (65% in clinical practice, 12% in research and 23% for both). Main reasons for not using OM were a lack of guidance/training on using and analysing OM, with 49% saying that they would use the tools if this was provided. 40% of those using OM in clinical practice used POS, and 80% used them to assess, evaluate and monitor change. The POS was also the main tool used in research, with the principle criteria for use being validation in Africa, access to the tool and time needed to complete it. Challenges to the use of tools were shortage of time and resources, lack of guidance and training for the professionals, poor health status of patients and complexity of OM. Researchers also have problems analysing OM data. The APCA African POS was the most common version of the POS used, and was reported as a valuable tool for measuring outcomes. Respondents indicated the ideal outcome tool should be short, multi-dimensional and easy to use.

**Conclusion:**

This was the first survey on professionals' views on OM in Africa. It showed that the APCA African POS was the most frequently OM used. Training and support are needed to help professionals utilise OM in palliative care, and OMs have an ongoing and important role in palliative care in Africa.

## Background

Within Africa the need for the provision of palliative care is great due to the high burden of disease. Life expectancy is just 53 years, ranging from 42 years in Zimbabwe to 63 years in Namibia, and under-five mortality is 142 per thousand live births [1]. By the end of 2009, there were 22.5 million people living with HIV and AIDS, with 1.3 million AIDS-related deaths in 2009 alone [2]. Cancer is also a major issue with 421,000 cancer-related deaths in 2008 and over 500,000 new cases in that year alone [3], with cancer rates set to increase dramatically over the coming years [4,5]. The age standardised mortality rate per 100,000 for the African region in 2004 was 1,945 [2].

Despite the above statistics palliative care provision across Africa is inconsistent [6] and research into palliative care, including end-of-life care in Africa is under-resourced and under-developed [7]. Yet it will not be possible to expand palliative care to the levels of coverage and quality needed across the region without robust evidence to influence policy, attract funds and change practice [8]. More palliative care research is happening on the continent now than ever before, and collaborations taking place in light of the "Venice Declaration" [9] which called for a focus on research in the development of palliative care in developing countries through collaborative partnerships. However, central to the ongoing development of research in palliative care in Africa, is the measurement of the effects and outcomes on patients [10]. Thus, there is a need for both the development of, and training in, the use of outcome measures in palliative care [11,12].

### Outcome scales

An outcome can be described as the change in a patient's current and future health status that can be attributed to preceding healthcare [13] and is usually defined in terms of the achievement or failure of interventions [14]. The measurement of health outcomes can be linked to the assessment of the appropriateness of health care interventions. Therefore the use of outcome measures can help determine whether a method of treatment or intervention is worthwhile [15].

Patient-reported outcome measurement (PROM) plays an increasingly important role in palliative care, and is used in clinical care (e.g. assessing the health status of patients in a hospital at admission), audit (e.g. quality assurance of services) and research (e.g. study the effectiveness of an intervention). The measurement of effects and outcomes on patients is important for quality improvement, needs assessment, and the evaluation of interventions whether in clinical practice or through specific trials [12]. Doctors and nurses have reported favorable experiences of outcome measurement use in palliative care [16].

A variety of PROMs are in use in palliative care [17-20]. Some of the commonest outcome measures used both for clinical practice and research include the Palliative care Outcome Scale developed in 1999 ((POS; http://pos-pal.org) [21,22], the APCA African POS developed in 2007 [23,24], the Support Team Assessment Scale (STAS) developed in 1986 [25], the Memorial Symptom Assessment Scale (MSAS) developed in 1994 [26], and the Edmonton Symptom Assessment Scale (ESAS) developed in 1991 [27].

Whilst there are a plethora of outcome measures that can be used in palliative care, there is a lack of outcome measures validated for the African setting [7,11,23]. The APCA African POS is the first outcome measure to be developed and validated within Africa. It was developed by a team of experts working across the region, and piloted in eight African countries (Botswana, Kenya, Malawi, South Africa, Tanzania, Uganda, Zambia and Zimbabwe) [23], following validation in five sites in South Africa and Uganda [24]. A similar tool for children - the APCA African Children's-POS (C-POS) is also currently under development. The Missoula-Vitas quality of life tool has also been adapted and validated for use in the Ugandan context [28], as has an HIV-related tool, the MOS-HIV [29]. Studies are also underway with regards to the usability of the Functional Assessment of Chronic Illness Therapy- Palliative Care Scale (FACiT-PAL) [30] in Uganda, Kenya and South Africa.

Since its development in 2007, the APCA African POS has been used widely across the region in both research and clinical practice, for example in a public health evaluation of palliative care in Kenya and Uganda [31], a situational analysis of palliative care in Namibia [32], an audit project in South Africa and Uganda [33]. However, little is known of the experiences of health professionals in using the APCA African POS, or any other outcome measures in the region.

### The survey

PRISMA (**"**Reflecting the **P**ositive dive**R**sities of European pr**I**orities for re**S**earch and **M**easurement in end-of-life c**A**re**") **was a three year project funded by the European Commission with a special focus on outcome measurement in palliative care [10]. The African Palliative Care Association (APCA) was one of the partners in the PRISMA collaboration. One of the work packages within PRISMA concentrated on the experiences of professionals using outcome measures in end of life care, through an online survey which was completed both in Europe and in Africa [12].

The aim of the survey in Africa was similar to that in Europe [12], to describe the practice of use of tools and outcome measures in different settings across Africa; to identify which tools are used in clinical care/audit and research; to describe the views of users regarding advantages and problems of using outcome measures; to describe the use and experiences with the POS; and to describe participants' views on further development of outcome measures in Africa.

## Methods

The survey methods are described in detail elsewhere [12]. A questionnaire was developed and piloted for the European online survey and was adapted to the African setting by including tools commonly used in Africa, in particular the APCA Africa POS. The questionnaire contained general questions on the use of tools, including a screening question to ascertain whether participants have used tools or not; whether and how often they use tools in clinical practice/audit or research, how they use the results, the advantages and disadvantages of using tools; issues with regards to further development of tools, and finally demographic information on the respondents themselves. An invitation letter was sent by email, stating that the survey was being undertaken by APCA in conjunction with the PRISMA team. Following completion of the online survey, results were collated by the Centre of Evaluation and Methods (ZEM) at the University of Bonn in Germany, who also hosted the survey.

Following the CHERRIES guidelines for reporting of web-based surveys [34], the participation rate was calculated dividing the number of replies of the first question by the number of unique site visitors (defined as those visiting the first page of the online survey) and the completion rate was calculated by the number of participants answering the first question divided by the number of people submitting the last question. Descriptive analyses of all questions were conducted reporting absolute numbers and frequencies for categorical data and means and standard deviation for continuous variables. As completion rates of answers varied throughout the questionnaire, we report the denominator of each answer separately. SPSS Statistics version 17.0 was used for quantitative analysis.

Ethical approval for this study was obtained from the Ethics Committee of the Ugandan National Council for Science and Technology (IS 62).

### Sample

Participants were drawn from the contacts database of APCA. Prior to sending emails inviting individuals to participate, individuals not working in Africa, as well as those known not to be clinicians, were removed from the list. A total of 422 invitation emails were sent in January 2010. Non-clinical respondents and those not involved in research or education were asked to say so that this information could be used for further data cleansing. Reminder emails were sent out in February 2010.

## Results

### Sample Characteristics

301 unique site visitors were counted on the first page (which gave more specific information about the survey) of the African survey. 168/301 respondents from 24 countries (see Table 1) replied to the first question (participation rate 56%) and 81/168 (48%) completed the survey. Eighty-eight respondents provided demographic information. 62% were female, average age was 47 years (SD 9) and 66% had more than 5 years of work experience in palliative care.

**Table 1:** Percentage of respondents from different countries (n = 76)

Percentage of Respondents	Countries
26	Uganda

16	Kenya

6	South Africa

3	Zimbabwe

3	Zambia

2 each	Lesotho, Swaziland, Rwanda, Nigeria, Cameroon

1 each	Botswana, Egypt, Ethiopia, Ghana, Morocco, Mozambique, Namibia, Sierra Leone, Tanzania, Tunisia,

2	Missing

### Use of OMs

60 people were not using outcome measures and they gave several reasons for this, including lack of guidance (15/60); lack of training on how to use tools (12/60) and how to analyse data (11/60); time constraints (10/60) and a lack of an outcome measure validated for the African setting (8/60). However, 49 stated that they would start using tools if more information and guidance was provided (24/49), appropriate tools were available (20/49), and more training was provided (18/49).

104 professionals used OM in clinical practice and research but only 78 gave more detailed information about their professional background. About two thirds had a clinical background and about a quarter had both a clinical and research background (see Table 2). Two thirds of respondents had more than 5 years of experience working in palliative care

**Table 2:** Professional background of those using Outcome Measures

Profession (n = 78)	Clinician Researcher Both	51 (65%) 9 (12%) 18 (23%)
**Clinician (n = 69)**	Physician Nurse Other	27 (39%) 29 (42%) 13 (19%)

**Researcher (n = 27)**	Medicine Nursing Psychology Social science Other	7 (26%) 5 (19%) 3 (11%) 7 (26%) 5 (19%)

The most common tool for measuring outcomes used in clinical practice was the POS (44/111); 29/111 used the Karnofsky Performance Status Scale (KPS), 26/111 the Palliative Care Assessment (PACA), 17/111 the PAL performance scale (PPS) and 16/111 the ESAS. These tools were used in clinical practice for a variety of reasons, the most common being assessment of patients symptoms, needs and problems (73/82), followed by using it to evaluate the effect of an intervention or service (65/82) or to monitor changes in patients' health status or quality of life (56/82).

Similar tools were also used in research, with 25/111 using the POS, 9/111 the PACA,8/111 the KPS and 6/111 the ESAS. The choice of tools used in research was influenced by whether it had been validated within palliative care (29/40), whether they had access to the tool (25/40) and the time needed for completion (23/40).

The most common tool used in both clinical care and research was the POS with 72/111 of respondents reporting to have used it. The APCA African POS was the most common version with 30/39 of respondents using it in clinical practice, and only 10/30 using the original POS, also referred to as POS Version one (http://pos-pal.org). In research, 21/25 used the APCA African POS and 9/25 the POS, with some having used both at some time.

### Reasons to stop using OMs

When asked why they had stopped using the POS or the APCA African POS, various reasons were given, including that they stopped using the POS as it was not validated in Africa, and started using the APCA African POS. When used in research, the tool is just used for the duration of that research, hence several participants had stopped using it as the research they were involved in had been completed. There was also a comment that measures need to be adapted for use by community volunteers.

### Use of the APCA African POS

In clinical practice, the APCA African POS was commonly used: to assess patients symptoms, needs and problems; to assess families needs; to monitor change in the patients health status or quality of life; to facilitate communication with the patient and their family; and to evaluate the effect of an intervention or the quality of care given. Questions on the APCA African POS about pain, symptoms, support, whether life is worthwhile and help for the family were seen to be the most useful questions, although all of the questions were seen to be useful in the measurement of care outcomes.

Respondents noted that the APCA African POS was seen as easy to use and enabled participants to get information from the patients about the services being provided and helped them to improve the care that they are giving. Generally respondents felt that the APCA African POS was user and client friendly, not cumbersome, was clearly laid out and specific, and includes both the family and patient which is important in the provision of palliative care. It also helped to improve communication between the health professionals, the patients and the family. One respondent commented that *"Using the APCA POS has helped facilities to use the information for patient care improvement - you are able to prepare the patient and the family on preparing for the future and also to ensure that the family builds up confidence in caring for the sick family member. To ensure that the family has enough information on the patients' illness."*

While there were advantages in using the APCA African POS, respondents also commented on challenges to its use. These included: family members not wanting to comment when asked about whether they had received help and advice to plan for the future as they interpret this question as though the patient is about to die; a lack of time to complete the tool; a lack of clear guidelines on interpreting the scores; language barriers; and the inability of some patients to complete the tool due to literacy and/or frailty. Generally however, respondents reported that their experience of using the APCA African POS had been a positive one, with 87% responding that their experience of using it was good or very good, 11% that it was neither good nor bad, and 2% that it was bad.

### Ideal tool properties

Despite the challenges above, there is a willingness to use outcome measures, and therefore to look at the outcomes of the care that they are providing, and to identify ways how these can be improved. When asked about their ideal outcome measure, the majority (73%) said that it should have between 6 and 15 questions, thus implying that it should be long enough to have meaning and measure the multi-dimensional aspect of palliative care, but short enough so that it is quick and easy to use and does not over burden the patients or staff.

## Discussion

This is the first survey reporting the views of African-based health professionals on the use and experiences of outcome measurement in palliative care.

It is evident that outcome measures are being used across Africa within the palliative care setting despite the fact that in 2005 there were no validated tools for use in palliative care in the region. In addition the importance of training and guidance on how to use outcome measures is recognised, and with increased support, more people would be able to use such measures both in clinical practice and research. Challenges do, however exist in the use of outcome measures. Some of the challenges identified in the survey included that the patients may be too frail, ill, or cognitively impaired to complete outcome measures, and have low literacy or educational levels and therefore need the support of staff to complete them. Staff do not have the time to use the measures, and many places are short staffed, and they may not know how to use them, or the importance of using them. Additionally, many of the tools are too complex, especially those intended for researchers, there is a lack of understanding in analysing what the results mean and their implications. Therefore there is a need for support in this area. Some of these challenges reflect those found in other studies, for example, Hughes et al in the UK in 2003 [35] found that key issues in the implementation of outcome measures for clinical practice included time constraints, staff work-load and training. These are issues that are important for professionals wherever they work, with the need for adequate training so that they understand the value of using such measures. Alongside this, the burden of outcome measures on the patients completing them needs to be addressed [36,37], and is particularly important within the palliative care context as some people are very sick and at the end of their lives.

Whilst it is encouraging to see that the APCA African POS is the most frequently used outcome measure in the region, this is not surprising as it is the only measure developed and validated for use in palliative care in Africa, and as this was one of the criteria used to choose an outcome measure it narrows the choices down. More significantly, it is encouraging that the number of questions in the APCA African POS (i.e. 10), falls within the range of questions of an ideal tool described by respondents. Whilst this survey demonstrates the use of OM's across the region, the use of such tools is not standardised locally and there is an ongoing need to train individuals on the use of tools such as the APCA African POS.

### Strengths and limitations of the survey

This survey reached professionals from a variety of medical, nursing and other professional backgrounds working in palliative care across a wide region. Using a web-based design allowed us to reach potentially more respondents than would have been possible should we have used a postal survey.

There are however, a number of limitations to the survey. First the sampling was conducted via the APCA contacts database which was heavily biased towards the East African region, as many people had signed up for membership during the APCA conference in Kenya, where there was a far greater number of individuals from East Africa than other parts of the region. Second, when people had expressed an interest in using the APCA African POS their names had been added to the database, thus the sample was potentially biased towards people interested in outcome measurement and POS users. Thirdly, the survey was conducted in English only, thus potentially preventing non-English speakers from completing the survey.

## Conclusion

This was the first survey addressing professionals' views on outcome measures in Africa. It was encouraging to see that a variety of tools are being used in clinical care and research in the region, with the APCA African POS being the most frequently used. Similar to within Europe, training and support is necessary and important to foster greater use of outcome measurement in palliative care in Africa. However, it was clear from the survey that outcome measurement has an ongoing and important role in palliative care in Africa.

## Competing interests

The authors declare that they have no competing interests.

## Authors' contributions

JD was involved in the conception and design of the survey, the acquisition of data and interpretation of results and drafted the manuscript. SS was involved in the conception and design of the survey, and analysis and interpretation of data. HB was involved in the analysis of data. BD was involved in the conception and design of the survey and analysis and interpretation of data. IJH was involved in the conception and design of the survey. RH was involved in the conception and design of the survey and the interpretation of results. CB was involved in the conception and design of the survey, and analysis and interpretation of data. All authors critically revised the manuscript and read and approved the final document.

**Table 3:** Use of the POS

Use of POS in Africa (incl. APCA African POS)	POS (%) (n = 71)	APCA African POS (%) (n = 72)
**Yes, currently using**	28	44

**Yes, but stopped**	30	24

**No**	42	32

## Pre-publication history

The pre-publication history for this paper can be accessed here:

http://www.biomedcentral.com/1472-684X/11/1/prepub
